# Asthma patients’ perception on their care pathway: a qualitative study

**DOI:** 10.1038/s41533-019-0121-2

**Published:** 2019-04-02

**Authors:** Anissa Hannane, Lilia Misane, Gilles Devouassoux, Cyrille Colin, Laurent Letrilliart

**Affiliations:** 1 Univ. Lyon, Université Claude Bernard Lyon 1, Collège universitaire de médecine générale, F-69008 Lyon, France; 2 Univ. Lyon, Université Claude-Bernard Lyon 1, Lyon, F-69008 France; 3 Hôpital de la Croix-Rousse, Service de pneumologie, Hospices Civils de Lyon, Lyon, France; 4 Unité d’Evaluation Médico-Economique, Pôle Information Médicale Evaluation Recherche, Hospices Civils de Lyon, 69003 Lyon, France; 5 Univ. Lyon, Université Claude Bernard Lyon 1, HESPER EA 7425, F-69008 Lyon, France

## Abstract

Because of insufficient asthma control in many patients, the collaboration between stakeholders is regarded as a promising strategy to improve asthma outcomes. This study explored the perceptions of French adult asthma patients on their care pathway. We conducted a qualitative study based on the interviews of 30 asthma patients aged 18–40 years, recruited in French primary care. We performed a thematic analysis of the data collected, using the NVivo software. According to the patients, the stakeholders involved in asthma management included those visible to healthcare professionals (patient, general practitioner, specialist(s), pharmacist, physiotherapist, family and friends) and those concealed by the patients (complementary and alternative practitioners); other stakeholders, such as nurses and occupational physicians, were not involved. Asthma management at diagnosis and follow-up phases proved to be unstructured, and were associated with poor patient education. This was supported by patients’ ambivalence (in relation to illness and treatments), poor communication between patients and healthcare professionals (lack of listening and use of inappropriate vocabulary by physicians, underreporting of alternative medicine use by patients) and weak cooperation between professionals (limited to interaction between the general practitioner and the specialist, either pulmonologist or allergist). Asthma management would probably benefit from a more coordinated care pathway at each phase of the disease that is consistent with the expectations and goals of the patients. It should be based on improved organization (involvement of other healthcare professionals and the patient as partners) and processes (regular follow-up, specific tools such as peak flow meter or action plan).

## Introduction

Approximately 4% of the world population in 2007 and 6.7% of the French population in 2006 were diagnosed with asthma.^1,2^ The prevalence of this disease is increasing and is probably underestimated.^3^ Despite effective treatment and international guidelines, 45% of asthma patients in Europe and 48% in France remain uncontrolled according to the Global Initiative for Asthma (GINA) criteria.^4,5^ In the USA, the prevalence of asthma exacerbations among persons with asthma, although in decline, remained above 50% in the 2000s.^6^ This results in overuse of care resources, including general practitioners’ (GP) urgent care visits, emergency room visits, and hospitalizations, and also in preventable mortality.^7^

Collaborative care of chronic disease can improve patient outcomes, increase healthcare professional satisfaction, and reduce healthcare costs.^8^ It occurs when multiple healthcare professionals from different professional backgrounds provide comprehensive services by working with patients, their families and careers, to deliver the highest quality of care.^9^ In asthma management, interprofessional collaboration, self-management and patient–physician communication are promising strategies to reduce symptoms and improve quality of life, which are implemented at various levels across the world.^10^ The French healthcare system promotes the primary–secondary care collaboration by assigning to GPs the role of care coordinator. Collaborative care further emerges in France through the development of the role of nurses in monitoring chronic conditions, such as type 2 diabetes, chronic obstructive pulmonary disease (COPD), cognitive impairment, and cardiovascular prevention.^11^

According to healthcare professionals, potential barriers to the optimal management of asthma can be related to the patient (misbelief), to the physicians (lack of time or resources), and to the complexity of the disease (misdiagnosis).^12,13^ Few qualitative studies on collaborative care of adult asthma patients have been published internationally, including two combined Australian studies reported by Cheong et al.^14,15^. The first study showed that asthma patients received limited multidisciplinary care and were not interested in it. This study focused on the roles of the GP, the respiratory specialist (pulmonologist or allergist) and the pharmacist, and did not consider other stakeholders.^14^ The second study identified various sources of health advice and support selected by patients, including professional and also personal and impersonal health connections.^15^ The aim of the present study was to explore the perceptions of French adult asthma patients on their care pathway.

## Results

This study recruited 30 asthma patients from 13 GP practices between December 2016 and January 2018 (Table 1). Each interview lasted between 12 and 60 min, with a mean duration of 32.6 min. Eleven eligible patients declined to participate in the study (response rate: 73%) for the following reasons: lack of time (*n* = 6), no response (*n* = 4), and the feeling to have nothing to say (*n* = 1).

**Table 1:** Participant characteristics

	Total (*N* = 30), *n* (%)
Mean age (years)	29.3
Gender	
Female	18 (60.0)
Male	12 (40.0)
Asthma control	
Well controlled	10 (33.3)
Partly controlled	13 (43.3)
Uncontrolled	7 (23.3)
Diagnosis period	
Childhood	24 (80.0)
Adulthood	6 (20.0)
Respiratory specialist(s) consultation	
Yes	7 (23.3)
No	23 (76.7)
Current smoker	
Yes	7 (23.3)
No	23 (76.7)
Living environment	
Rural	19 (63.3)
Urban	11 (36.7)
Atopy	
Yes	18 (60.0)
No	12 (40.0)
Comorbidities	
Yes	6 (20.0)
No	24 (80.0)
Medical fee exemption status for low income	
Yes	3 (10.0)
No	27 (90.0)

### The stakeholders

#### Healthcare professionals

Patients considered the GP as the primary source of therapeutic and preventive care, and as the professional coordinating their care (Table 2). S/he was frequently involved in the differential diagnosis, in treatment initiation and adaptation, and in the assessment of side effects and adherence to treatment, as well as in the management of asthma comorbidities. Possible limitations of GPs included lack of provision of technical investigations and insufficient training to manage severe asthma. The specialist, either a pulmonologist or an allergist, had an important role as expert in asthma. S/he was often involved in the positive and etiological diagnoses, based on investigations such as spirometry and allergy tests, in the initial prescription of drugs, and in the follow-up of severe or uncontrolled asthma. Some patients felt that they received better management from the specialist owing to technical support, which can be provided during the follow-up or performed at critical moments such as pregnancy or resumption of sport. Patients attended a hospital emergency room when their GP was not available, or their symptoms were severe.

**Table 2:** The stakeholders (illustrative quotes)

Themes	Subthemes	Quotes
Healthcare professionals	General practitioner	P4: “And the doctor actually plays a key role because he’s here to make a diagnosis. […] I think that’s also him who has to see if it is getting worse or not.”
		P5: “The GP will focus more on something global.”
		P18: “I think he could check the breath and all that. I think he would be able to do it. But has he the equipment, that’s the question.”
	Specialists	P15: “A pulmonologist has been able, him, to perform the tests and then to give me an appropriate treatment.”
	Pharmacist	P17: “They know drugs a little better, a little more than doctors. That’s their job.”
	Occupational physician	P11: “As a result, when we come before the occupational physician, we rather tend to say that everything is going well to avoid any restriction.”
	CAM practitioner	P8: “Allopathy, in my view, is only symptomatic. […] The process that is put in place upstream, it’s more in soft medicine that I will find it.”
Patient	Experience	P1: “Everyday, actually, we learn to breathe that way; for us, it is no longer a discomfort.”
		P9: “I’m not saying that I’m asthmatic but… it occurs periodically”
		P16: “Anyway, I’m condemned.”
		P5: “I see the doctor when I really have to, because [otherwise] it’s a waste of time for everyone.”
		P26: “I saw some great professors, but nobody really knew how to cure this asthma.”
	Role	P4: “I am the first to be involved [in the management of asthma]!.”
	Difficulty	P14: “Unfortunately, I don’t know how to use them well [the inhalers] because I was not well trained.”
Other stakeholders		P7: “My mother is asthmatic, so she has the same treatments. Therefore, I’ve already seen her doing it [using inhalers].”
		P23: “That can be interesting to know… how to create a group of asthma sufferers like me.”

*CAM* complementary and alternative medicine

The roles of other healthcare professionals were perceived as smaller in the management of patients’ asthma. The pharmacist was involved in treatment dispensation and therapeutic education, especially targeting inhaler handling. S/he could participate to continuity of care for some patients, by extending drug dispensation beyond the physician’s prescription. The physiotherapist provided respiratory physiotherapy and sometimes contributed to education on inhaler handling. The occupational physician, when available at the workplace, was never involved in asthma management, even if the patient’s asthma was related to his or her professional activity. Whereas some patients reported a lack of confidence in the occupational physician, others considered that s/he could counsel them on the influence of their professional activity on their asthma.

Patients consulted other stakeholders including psychologists or professionals practising various kinds of complementary and alternative medicine (CAM) such as homeopathy [medicine based on the doctrine of *like cures like*], herbal medicine, sophrology [self-help method to manage stress and discover mindful living], osteopathy [medicine based on physical manipulation of muscle tissue and bones], acupuncture [component of traditional Chinese medicine in which thin needles are inserted into the body], and applied kinesiology [technique based on muscle testing]. These approaches were sometimes recommended by the pharmacist or the patient’s family, and occasionally the GP prescribed homeopathic medications. The main motivation of patients to consult these stakeholders was to obtain a treatment for the cause of asthma, especially targeting allergy, not just a treatment focused on the respiratory system. These therapies were used occasionally and perceived as softer than continuous conventional care. Patients expected more information from their physicians on these alternative forms of care.

#### Patients

Most patients accepted or were resigned to their asthma disease status, either by habit (accustomed to living with symptoms), by relativism (compared to other chronic diseases), or by fate. Asthma diagnosed during childhood or manifesting with symptoms considered acceptable favored this attitude. Patients tended to trivialize or even deny their disease, its chronicity, and its symptoms. Some of them underestimated the risk of exacerbation or even of death in case of severe asthma. Patients expressed contradictory statements regarding asthma management. On the one hand, they often showed passivity and lack of interest, associated sometimes with feeling guilty. They considered the disease as asthma exacerbations and therefore only managed symptoms. Their level of investment was related to the duration of asthma rather than to its seriousness. On the other hand, they wished to get involved in their care and even to become the main stakeholder. In particular, they had their own goals, both in terms of quality of life and prevention of serious exacerbations. They thought they could assess the severity of their symptoms, self-manage their treatment, and consult only when considered necessary. They also, however, recognized they had limited skills and knowledge to assess changing symptoms, to manipulate their therapeutic devices (especially due to the various types of inhalers), and to manage an exacerbation, due to a lack of education received from healthcare professionals. They reported acting on their environment to limit factors triggering exacerbations, even if it could excessively impact their daily life. For example, some patients confined themselves at home during periods of severe atmospheric pollution, and others moved house to limit exposure to allergens.

#### Other stakeholders

When the diagnosis of asthma was made during childhood, the parents participated in patient care by accompanying the child in the process of empowerment and providing moral support. When someone among family or friends also suffered from asthma and was able to share his or her experience, this represented a valuable resource for the patient. Other patients reported willingness to participate in patient groups to obtain education and to exchange knowledge on the subject, but they lacked information on such organizations.

### Patient relationships with healthcare professionals

#### Various types of relationships according to asthma phases

Patients experienced a rather paternalistic attitude from physicians during the diagnosis and treatment initiation phases (Table 3). Not being provided explanations as to the disease and the treatments was a source of frustration for some patients, while others found some comfort or even benefit to be directed by a physician, particularly for smoking cessation. Patients reported a more open relationship during the follow-up, especially regarding treatment adaptation. They were more at ease to give feedback on their experience regarding treatments effectiveness and side effects, and to express their willingness to maintain or discontinue them. Nevertheless, most patients reduced or even discontinued their treatments of their own accord when they felt an improvement in the course of their asthma. Overall, patients perceived specialists as more paternalistic than GPs. The practice or recognition of CAM therapies, especially homeopathy, was important to choose their GP.

**Table 3:** Relationships with healthcare professionals (illustrative quotes)

Themes	Subthemes	Quotes
Various types	Paternalistic approach	P1: “In fact, the pulmonologist, she was clear: « if you want to breathe better, that’s it. » A little closed on that, there was not much discussion to have.”
		P12: “In fact, I don’t discuss it. When I am prescribed something, well, I take it.”
		P23: “[what I would have liked during follow-up is that my GP] to have told me: « Well, OK, you’re asthmatic, that’s how it’s going to be. [Here are] all the steps you will go through to check that everything is fine. » Rather that than she takes care of me.”
	Shared decision-making	P5: “I like that doctors leave to the patient, [..] the appropriation of the disease and the treatment.”
		P20: “I continue Airomir^®^ [salbutamol]. But later maybe I’ll stop… It depends… I will see the GP so that he explains how to do so.”
		P8: “I have the advantage to have a homeopathic doctor, so it’s true that we have discussed from time to time to use homeopathy without ever really removing the long-term treatment that reassures me.”
Communication	Failure	P2: “But I have not been explained things so much. But me, I need to understand.”
		P2: “I have experience of other specialist physicians and I do not find that they know how to explain things and to listen to their patients.”
		P22: “Can asthma be controlled? Is it possible not to have it any more?”
	Unsaid	P14: I have never dared to ask [how to use treatments], by shyness.”
Dissatisfaction	Physicians	P2: “The regular physician had no blinkers; this is not the case for all [other physicians], I think.”
	Disease control	P16: “I will be really satisfied the day when I will be told: «That’s it! We have found a cure. »”
	Lack of recognition	P1: “I expect that she [my GP] takes my asthma seriously, because a substitute physician did not care whether I felt bad or not.”

#### Poor communication overall

Patients reported a lack of listening and answers to their questions. In addition, the vocabulary used by physicians was not always understandable to them. For example, many patients did not understand the notion of asthma control correctly; some thought that this referred to management of exacerbations, and others that this reflected to have been cured of the disease. They regretted that physicians focused on treatments and investigations rather than on patient experience. They viewed CAM practitioners as an opportunity to compensate for this poor communication and caring for them holistically. In parallel, many patients frequently did not express their feelings and did not ask their questions during the consultations with the physicians, either by habit, shyness, fear, or lack of interest.

#### Several causes of dissatisfaction

Most patients had confidence in the healthcare professionals, especially in their GP, who knew them best. The confidence of some patients was directly related to the perceived level of control of their asthma. They could then be dissatisfied and blame their GP if they felt their disease was poorly controlled, which they attributed to a lack of clinical examination, investigations, or referral to a specialist. They were also frustrated if they thought that their asthma was not medically and economically recognized as a chronic condition, either by caregivers or by the national health insurance system (especially with fee exemption status).

### Interprofessional collaboration

#### Patient experience

Patients attributed a significant role to the GP in interprofessional coordination, as s/he could refer them to other stakeholders, in particular to specialists, when judged necessary (Table 4). S/he was involved in this process from suspected asthma to its management. The complementarity between the GP and the specialist(s) was valued by patients to optimize diagnosis and treatment, especially in cases of severe asthma. However, such distribution of roles was not clear to all patients. Some of them did not find it useful to be referred to a specialist, whereas others regretted not having been referred early enough or not obtaining regular follow-up with a specialist. In particular, some patients contested the gatekeeping role of the GP, perceived as a barrier for accessing the allergist. In addition, other patients expected to be easily delivered short acting bronchodilators by the pharmacist when the prescription was ending. Patients did not consider pharmacists and occupational physicians as partners within their care pathways. They also reported that they concealed the intervention of CAM practitioners, and their therapies, from other healthcare professionals, mainly because they feared their judgment. Some patients did not feel concerned at all by the lack of care coordination, either because they considered their asthma not serious enough or because they wished to coordinate their care by themselves.

**Table 4:** Interprofessional collaboration (illustrative quotes)

Themes	Subthemes	Quotes
Experience	GP–specialist partnership	P12: “When I went to the pulmonologist, it was on a doctor’s recommendation, so they should have communicated together.”
		P16: “And then after a while, she [the pulmonologist] sends me back to my GB. Because, for her, I was not really a serious case, so… I think she actually only keeps serious cases.”
		P22: “It would be good in the management of the medical follow-up that indeed there is a real communication between physicians. Is it possible and is it done, I don’t know. But that would be good.”
		P5: “For me, it does not matter if it returns or it does not return [the communication between physicians]. In fact, it’s me who takes care of my health. So as long as I’m informed … Let’s say that I am the person responsible.”
	CAM practitioners exclusion	P8: “The osteopath, if he has something to say, he will generally walk on eggshells because… there are very few physicians able to hear it. It’s very compartmentalized.”
		P2: “I expect to be able to speak about that kind of treatment [CAM], which is not regarded as such by most of the medical profession, but which for me seems more than enough.”
Benefits		P24: “Group work is to be favored, because it allows to have several views.”
		P5: “I imagine that each time all test results are sent to the GP. That’s the principle.”
		P25: “We have a patient interpretation of what the doctor has told us, and when we have to forward the information, sometimes we do not forward everything or we have forgotten part of it or we have misunderstood. And the fact that they directly communicate actually avoids these unintentional omissions and oversights.”
Limitations		P20: “I don’t know. I think they do not really have time. Or they do not take time to do it.”
		P20: “I have never been asked who was my pulmonologist or who was my GP; so no, they do not communicate.”
		P28: “A client record that would be accessible to all healthcare professionals, so that each professional can follow the records.”

*GP* general practitioners, *CAM* complementary and alternative medicine

#### Perceived benefits and limitations

According to the patients, interprofessional coordination was essential to share information and provide them with the most appropriate follow-up. They considered that their GP had to be informed of all consultations, investigations, and treatments, regarding conventional care for asthma or any other condition. They expected extending the coordination process to other stakeholders such as pharmacists and CAM practitioners, in order to achieve true interdisciplinary management. The various ways of communication used between the GP and specialist(s) were postal or electronic mail, telephone, or through the patient (via paper mail or oral information). The main perceived barriers to coordination were the lack of physician time to communicate and the risks regarding confidentiality. Patients could also face difficulties to transfer their medical record, in case they had to change their physician. They suggested the use of a shared electronic health record to improve communication between all healthcare professionals.

## Discussion

**Fig. 1:**
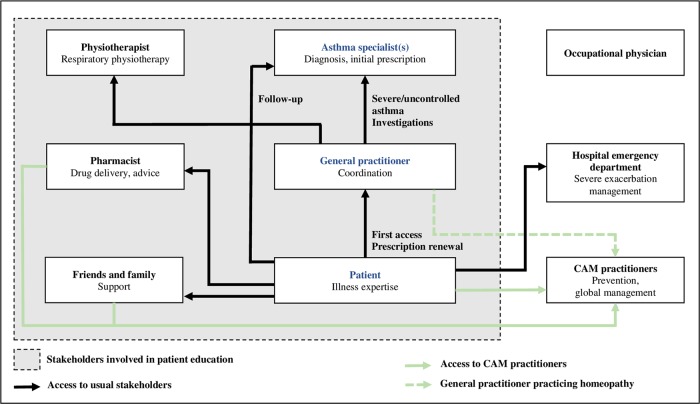
Modeling of current asthma patient care pathway and stakeholders’ roles

Apart from patients, the main stakeholders involved in the management of asthma were the GP and the specialists (pulmonologist or allergist), who had a role in its diagnosis, its follow-up, and patient education. The pharmacist and the occupational physician, when involved, had a limited role, whereas CAM practitioners represented a significant resource for patients. Most patients had insufficient knowledge of asthma, trivialized their disease, and adopted a passive attitude, although some of them tended to become more autonomous in their care. Relationships between patients and healthcare professionals varied according to the disease phases, from paternalistic to patient-centered, but communication remained poor overall. The GP was the main referent, coordinating patient care, and interprofessional collaboration; when this existed, it was limited to that between the GP and specialist. Among the various other stakeholders, only the pharmacist, the physiotherapist, and the patient’s friends and family participated in patient education, without collaboration. The delineated patient care pathways and the current stakeholders’ roles are modeled in Fig. 1.

Patient behavior reflects the unstructured management of asthma over its different phases. For many patients, the diagnosis of asthma remained uncertain. Its diagnosis and follow-up were not based on the results of spirometry, as described in other countries.^16^ Patients mainly consulted during symptom exacerbation, which reduces the renewal of their prescription to an emergency service.^17^ This discontinuous care leaves little room for physicians to have an educational approach integrated into consultations. In addition, patients rarely accessed available supported self-management programs because they were unfamiliar with them, despite their proven effectiveness on reducing emergency care and hospitalizations but also in improving quality of life.^18^ The lack of structuring of patients’ care pathways fed into their ambivalence towards asthma diagnosis and ongoing management, poor communication between patients and the healthcare professionals, and weak cooperation between them.

Patients’ desire for recognition of their disease status contrasted with their trivialization of the disease. Paradoxically, the daily administration of alternative therapies or salbutamol appeared to them less constraining than that of their long-term treatment. Such patient ambivalence represents a step in the process of behavior change towards asthma, which can lead to therapeutic adherence and self-management. This process should be supported by healthcare professionals, as part of an interaction with the patient based on motivational interviewing, which includes listening, support, and positive strengthening.^19^

Poor communication, in terms of form and content, limits therapeutic adherence and favors patient attitudes of disinterest or denial.^20^ A better understanding of patients’ lifestyle and bio-psycho-social status by healthcare professionals is required to set an educational diagnosis based on patients’ personal goals.^21^ For example, the physician and the patient must find an acceptable compromise for long-term treatment, which can be considered as necessary by physician but poorly accepted by the patient.^22^ However, physician communication is often disrupted because of the episodic course of the consultations and underreporting by the patients.^23^ As shown in our study, the use of CAM is frequently concealed from healthcare professionals, which is in agreement with data from Australia and the USA.^24^ The explanation invoked by the patients in the present study, namely the lack of openness of the doctors, corroborates findings from this review. In fact, physicians declare themselves uncomfortable with this issue and avoid talking about it with patients.^25^ Herein, patients using CAM wished to discuss this with their physicians, and even to get informed by them about these practices.

Patients considered that their GP treated them overall, which is particularly important when he/she is the only professional consulted; this has previously been observed among Australian asthmatic patients.^14^ The situations of interprofessional collaboration reported by the patients were rare and only concerned that between the GP and the specialist (pulmonologist or allergist); the GP playing the role of coordinator. Referral to the specialist seemed limited to the needs for investigations or for the management of severe asthma. According to a Brazilian study, a more formal collaboration between primary and secondary care could improve quality of asthma care^26^. Patients limited the role of the pharmacist to treatment dispensation, as observed in previous studies.^15,27^ However, the pharmacist’s greater involvement in patient education and treatment review could improve asthma symptoms and adherence to treatments.^10,28^ According to the patients, the existence of asthma and possible contributing factors were not systematically searched for by the occupational physician nor declared by the patient, especially by fear of being discriminated against. Greater involvement of the occupational physician, in collaboration with other healthcare professionals, could yield a better diagnosis of occupational asthma, which is often under-diagnosed,^29^ and to a better management of severe or uncontrolled asthma.^30^ Asthmatic patients using CAM are likely to be less adherent to inhaled corticosteroids^31^ and less well controlled^32^ Consulting CAM practitioners is motivated by the search for the cure of the disease and a holistic management (conventional medicine being considered ineffective), and by the fear of the side effects and constraints of treatments.^33^ Patients are satisfied with CAM,^24^ despite the lack of evidence for effectiveness beyond the placebo effect in asthma management.^34^ CAM practitioners are unable to collaborate with the physicians because they remain invisible in patients’ care pathway, as they believe that most physicians would not recognize them as legitimate practitioners. No patient cited nurses as actual or potential asthma care providers. However, the involvement of nurses in collaboration with GPs to manage asthma is reported to improve patient satisfaction and decrease hospitalization rate, as compared to usual care provided by GPs alone.^35^ Some patients wished to coordinate their care pathway themselves by including the professionals of their choice, including CAM practitioners, while others considered that they were even able to manage their asthma without the intervention of healthcare professionals. A proportion of French asthma patients interviewed in the present study expected interprofessional collaboration in their care, contrary to asthma patients in Australia, who are not personally interested in it.^14,15^ This difference may be due to specific gatekeeping rules; a patient can access a specialist if referred by his/her regular GP in France, but by any GP in Australia.^36^

The upper age limit of 40 years aimed to minimize the risk of including non-asthmatic patients (in particular COPD and asthma-COPD overlap syndrome),^37^ as asthma diagnosis is frequently based on clinical examination alone, without spirometry.^38^ This choice limited the inclusion of asthma patients with multimorbidity, who may have more interprofessional care needs. Although the sex ratio was unbalanced (60% women), it is consistent with the proportion of 62% observed in European asthma adults.^4^ The study sample contained more patients with well-controlled asthma than observed in the European asthma population (33% versus 20%)^4^, although patients with poor asthma control have more ambulatory care than well-controlled patients.^39^ Both patients mainly managed by a specialist or those who rarely consult healthcare professionals may be underrepresented herein, and it is of note that for these patients, interprofessional collaboration is likely to be rarer than for other asthma patients. Data saturation was reached after 22/30 interviews. One possible limitation of the interviews is the social desirability bias, which drives individuals to answer in a way that makes them look more favorable to the interviewers, as the recruitment took place in general practice and the interviewers were medical students.

Asthma management would probably benefit from a more structured follow-up, based on improved processes and organizations. A more objective positive and etiological diagnosis could facilitate its acceptance by the patient.^20^ Thereafter, patients should benefit from regular follow-up consultations, including the use of specific tools such as a peak flow meter or spirometry,^40^ standardized questionnaires for asthma control, and written action plans^41^ to improve patient adherence. Patient education, especially regarding self-management, should be systematically provided during the follow-up, either by a network of stakeholders or by referring to a dedicated service.

**Fig. 2:**
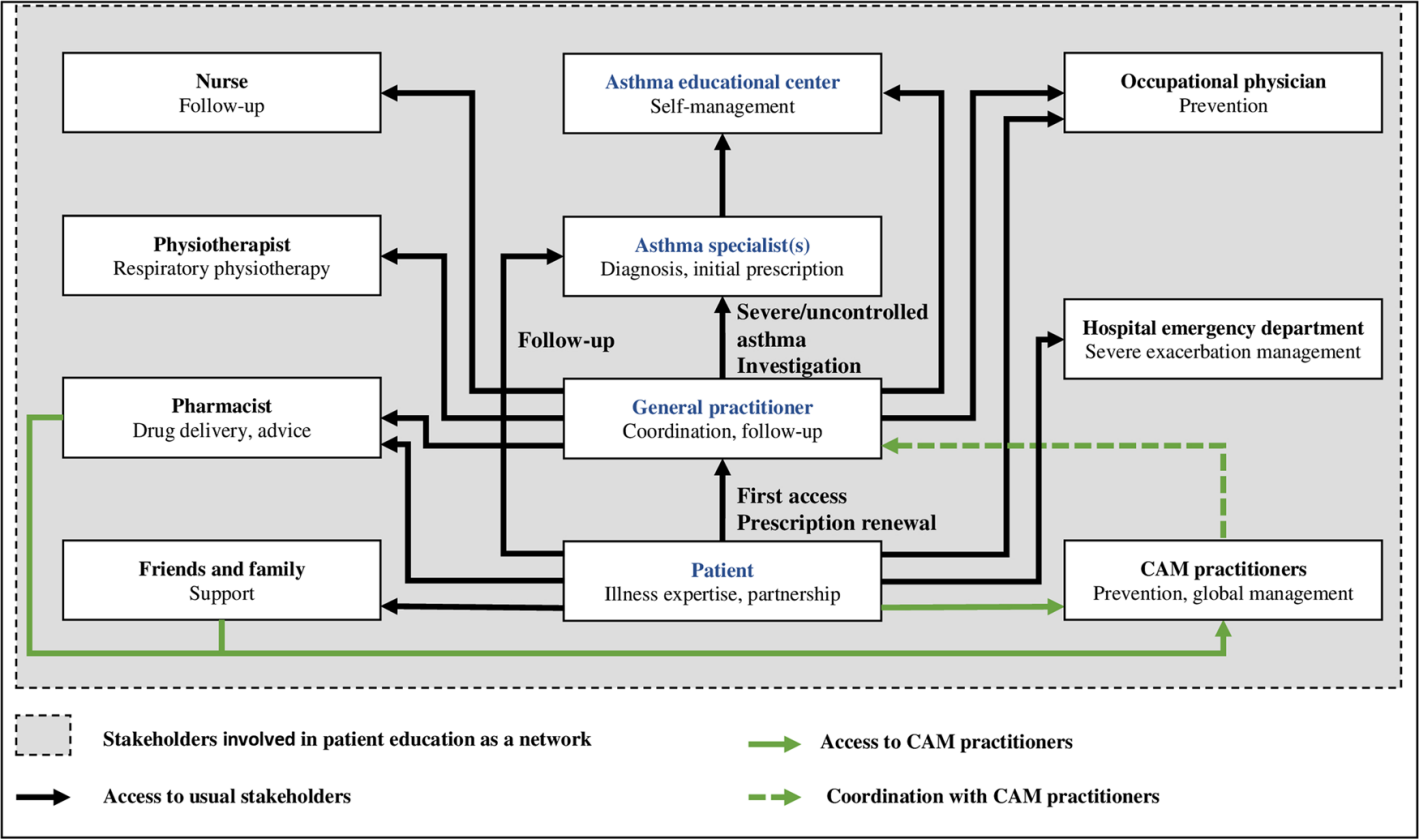
Modeling of expected asthma patient care pathway and stakeholders’ roles

Disease-management programs have become a reference strategy in chronic disease, to improve healthcare quality and to limit healthcare costs. In asthma management, current evidence is encouraging but not sufficient to recommend any particular intervention.^42^ In several countries, asthma disease-management programs integrate nurses, particularly to educate patients and perform respiratory tests,^10^ but not in France.^43^ In the UK, the asthma workforce includes respiratory nurse specialists usually located within acute environments.^44^ It is likely that a patient-as-partner approach, in which the patient is a full member of the healthcare team, could be more appropriate for asthma management than the traditional paternalistic approach, and even than the patient-centered approach.^45,46^ Based on our findings, we have developed an ideal transitional model for future care (Fig. 2). It incorporates the roles of all stakeholders, including educational structures, and reinforces the place of the patient as a partner in asthma collaborative care. Although an electronic health record shared between stakeholders, including the patient, has the potential to support care coordination, we are not aware of any assessment of its effectiveness in improving asthma collaborative care.^47^

Further research should focus on the views and expectations of the participating stakeholders identified in this study on asthma care coordination. As our study is relatively small and exploratory, it would be useful to supplement our findings and validate our model with observational data on the actual care pathways of asthma patients in various countries.

In conclusion, the management of French asthma patients is poorly structured. The interprofessional collaboration, currently limited to that between the GP and specialist, should be extended to other professionals, including pharmacists, nurses, and occupational physicians. This extended healthcare team should consider the expectations and goals of the patient and recognize him/her as a partner. Such development should be supported by appropriate processes, including a regular follow-up and specific tools such as peak flow meter or action plan, to provide patients with more accessible and personalized education and improve the quality of asthma care. Healthcare professionals should be able to discuss CAM involved in the patient care pathway.

## Methods

We conducted a qualitative study based on semi-structured interviews and according to the grounded-theory approach, based on inductive gathering and analysis of data.^48^

### Patient recruitment

We included patients aged 18–40 years with a diagnosis of asthma, whether registered in the patient’s record or self-reported, provided that they were prescribed at least three asthma medications at three different dates. The age limit was defined to prevent inclusion of patients with COPD.^37^ We excluded patients with an associated COPD diagnosis, poor fluency in French, and a legal protection status. Patients were included until data saturation was reached. The participants were recruited in GP practices from the Rhône-Alpes region of France according to a purposeful sampling strategy. We aimed to obtain a diverse sample of patients in terms of age, gender, living environment, and asthma control. Eligible patients were identified by two of the authors (A.H. and L.M.) in various voluntary GP practices, based on indications from the GP holding the practice and searches in the electronic health records.

### Data collection

Participants were interviewed face-to-face by one of two authors (L.M. or A.H.), in a place of their choosing. The interviewers had been trained to conduct an interview and to develop reflexivity. An interview guide was developed based on a review of the literature and on discussion between the authors. The guide explored patient perception of the asthma disease, its diagnosis, treatment, current and expected care, and collaborative management (Appendix 1). It was improved based on the findings of the first interviews. Each interview was audio-recorded. We also collected information on participant characteristics (demographics, asthma control according to the GINA criteria,^49^ diagnosis period, respiratory specialist(s) consultation, smoking status, living environment, atopy, comorbidities, fee exemption status) using a questionnaire filled-out by the participants.

### Data analysis

Interviews were fully and anonymously transcribed verbatim and analyzed using the NVivo 11 Pro software.^50^ After a full reading of the transcripts, two authors (L.M. and A.H.) analyzed and coded their content, based on regular discussions. The concepts identified through the open coding were classified according to axial coding and reviewed by a third author (L.L.). The resulting categories were gathered into the three main themes structuring the results section, according to a selective coding process.

### Ethical aspects

The ethics board of the Collège Universitaire de Médecine Générale of the University of Lyon 1 granted approval to the study (IRB 2017-09-05-03). In addition, this study was declared to the French Data Protection Authority (CNIL, 2016-10-27). A written consent was obtained from each participant, after s/he had been informed of the aim and design of the study.

### Reporting Summary

Further information on research design is available in the Nature Research Reporting Summary linked to this article.

## Supplementary information

Appendix 1

Life Sci Reporting Summary

## Data Availability

The data that support the findings of this study are available from the corresponding author on reasonable request.

## Supplementary information

**Supplementary information** accompanies the paper on the *npj Primary Care Respiratory Medicine* website (10.1038/s41533-019-0121-2).
