# Supplementary material for: Asthma patients’ perception on their care pathway: a qualitative study
Source: NPJ Prim Care Respir Med. 2019 Apr 2;29:9. doi: 10.1038/s41533-019-0121-2 (PMC6445145; doi:10.1038/s41533-019-0121-2)
Supplement: Supplementary file 1 — Appendix 1 [file 41533_2019_121_MOESM1_ESM.pdf]

Appendix 1 Interview guide

Introduction

- For you, what is asthma? (How does it manifest? What is asthma exacerbation?)
- How do you experience your asthma? (Give the example of a typical day)
- According to you, are there risks associated to asthma?
- According to you, what is causing asthma?
- Do you have question regarding your asthma? (Did you find answers? If yes, how?)

Diagnostic

- How was your asthma diagnosed? (By whom? How? When did your first consult a physician and for which reason or which worry? What was the role of your regular physician?)
- When asthma has been mentioned for the first time, what was your feeling?

Treatment

- What are your treatments for asthma? (Specify the names. What are they for? Who prescribed them to you?)
- Could you discuss the choice of the treatments?
- Did you learn to handle the various drug devices? (If yes, how? If no, who do you think could help you?)
- Do you take your treatments as prescribed by the physician? (If not, why?)
- Did you use other treatments?

Follow-up

- Do you think that your asthma is well controlled?
- What do you do if your asthma gets better or worse?

Collaborative care

- Who is involved in the management of your asthma, whether healthcare professional or not? (what is the role of each one? Is your occupational physician aware of your asthma and has he a role? Does the pharmacist have a role?)
- Do those involved communicate together? (How and why? If not, do you think that they should and why?)
- Who is the main healthcare professional in charge of managing your asthma?

Conclusion

- Are you satisfied of the management of your asthma?
- What are your expectations regarding your care for asthma?
- Are there other issues that we did not mention and that you would like to speak about?
